# Iterative gene integration mediated by 26S rDNA and non-homologous end joining for the efficient production of lycopene in *Yarrowia lipolytica*

**DOI:** 10.1186/s40643-023-00697-6

**Published:** 2023-11-24

**Authors:** Zhen Luo, Jiang-Ting Shi, Xin-Liang Chen, Jun Chen, Feng Liu, Liu-Jing Wei, Qiang Hua

**Affiliations:** 1 State Key Laboratory of Bioreactor Engineering, East China University of Science and Technology, 130 Meilong Road, Shanghai, 200237 People’s Republic of China; 2 Shanghai Collaborative Innovation Center for Biomanufacturing Technology, 130 Meilong Road, Shanghai, 200237 China

**Keywords:** *Yarrowia lipolytica*, Lycopene, Iterative gene integration, Lipid, Fermentation

## Abstract

Because of its potent antioxidant effects, lycopene has been used in various industries including, but not limited to, food, medical, and cosmetic industries. *Yarrowia lipolytica*, a non-conventional yeast species, is a promising chassis due to its natural mevalonate (MVA) pathway, abundant precursor acetyl coenzyme A content, and oleaginous properties. Several gene editing tools have been developed for *Y. lipolytica* along with engineering strategies for tetraterpenoid production. In this study, we engineered *Y. lipolytica* following multi-level strategies for efficient lycopene accumulation. We first evaluated the performance of the key lycopene biosynthetic genes *crtE*, *crtB*, and *crtI,* expressed via ribosomal DNA (rDNA) mediated multicopy random integration in the *HMG1*- and *GGS1*-overexpressing background strain. Further improvement in lycopene production was achieved by overexpressing the key genes for MVA synthesis via non-homologous end joining (NHEJ) mediated multi-round iterative transformation. Efficient strategies in the MVA and lipid synthesis pathways were combined to improve lycopene production with a yield of 430.5 mg/L. This strain produced 121 mg/g dry cell weight of lycopene in a 5-L fed-batch fermentation system. Our findings demonstrated iterative gene integration mediated by 26S rDNA and NHEJ for the efficient production of lycopene in *Y. lipolytica*. These strategies can be applied to induce *Y. lipolytica* to produce other tetraterpenoids.

**Graphical Abstract:**

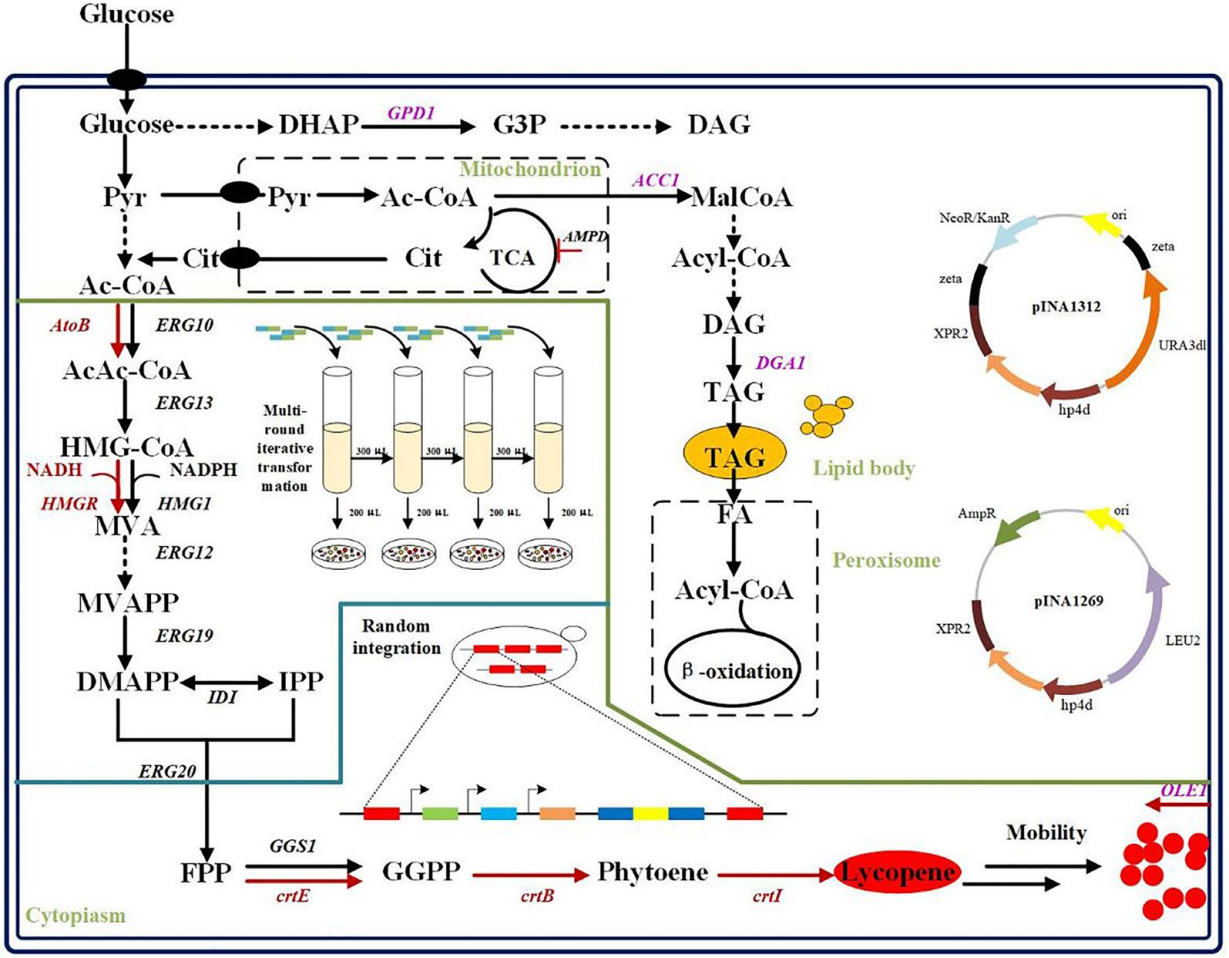

**Supplementary Information:**

The online version contains supplementary material available at 10.1186/s40643-023-00697-6.

## Introduction

Lycopene, belonging to the tetraterpenoid family of terpenoids, is an excellent antioxidant with antiaging and anti-cancer properties (Aghajanpour et al. 2017; Jhou et al. 2017) and therefore has a high market value in various industries including food, medical, and cosmetics. Lycopene is extracted from plants or synthesized either chemically or via microbial fermentation; however, extraction from fruits, such as tomatoes, via conventional strategies is associated with low yield (Shi and Le Maguer 2000). Meanwhile, chemical synthesis is limited because of the presence of chemical residues and strict reaction conditions (Michael McClain and Bausch 2003). In comparison, biotechnological production provides a cost-effective and sustainable approach. Advancements in metabolic engineering and synthetic biology have facilitated the production of lycopene via myriad microbial hosts, including *Escherichia coli* (Wu et al. 2017), *Saccharomyces cerevisiae* (Shi et al. 2019), *Yarrowia lipolytica* (Luo et al. 2020), and *Pichia pastoris* (Araya-Garay et al. 2012). The highest lycopene content synthesized to date, 448 mg/g of dry cell weight (DCW), was obtained from engineered *E. coli* (Coussement et al. 2017). Further, a lycopene titer of 6.03 g/L was obtained from *S. cerevisiae* in a 70-L bioreactor (Wang et al. 2020), whereas 4.2 g/L and 17.6 g/L of lycopene were achieved from engineered *Y. lipolytica* (Luo et al. 2020; Ma et al. 2022).

As an unconventional oleaginous yeast, *Y. lipolytica* is used in foods and medicine as it is “generally regarded as safe” (Nicaud 2012), and its natural mevalonate (MVA) pathway allows it to generate abundant precursor acetyl coenzyme A (acetyl-CoA) (Ma et al. 2021), facilitating production, and accumulation, of various terpenoids. Moreover, large amounts of lipid droplets that store lipophilic terpenoids, such as lycopene, accumulate in *Y. lipolytica*. Importantly, *Y. lipolytica* can utilize low-cost feedstocks to obtain high-value products (Mirończuk et al. 2016; Nogué et al. 2018); therefore, it is a productive host for efficient lycopene biosynthesis.

**Fig. 1:**
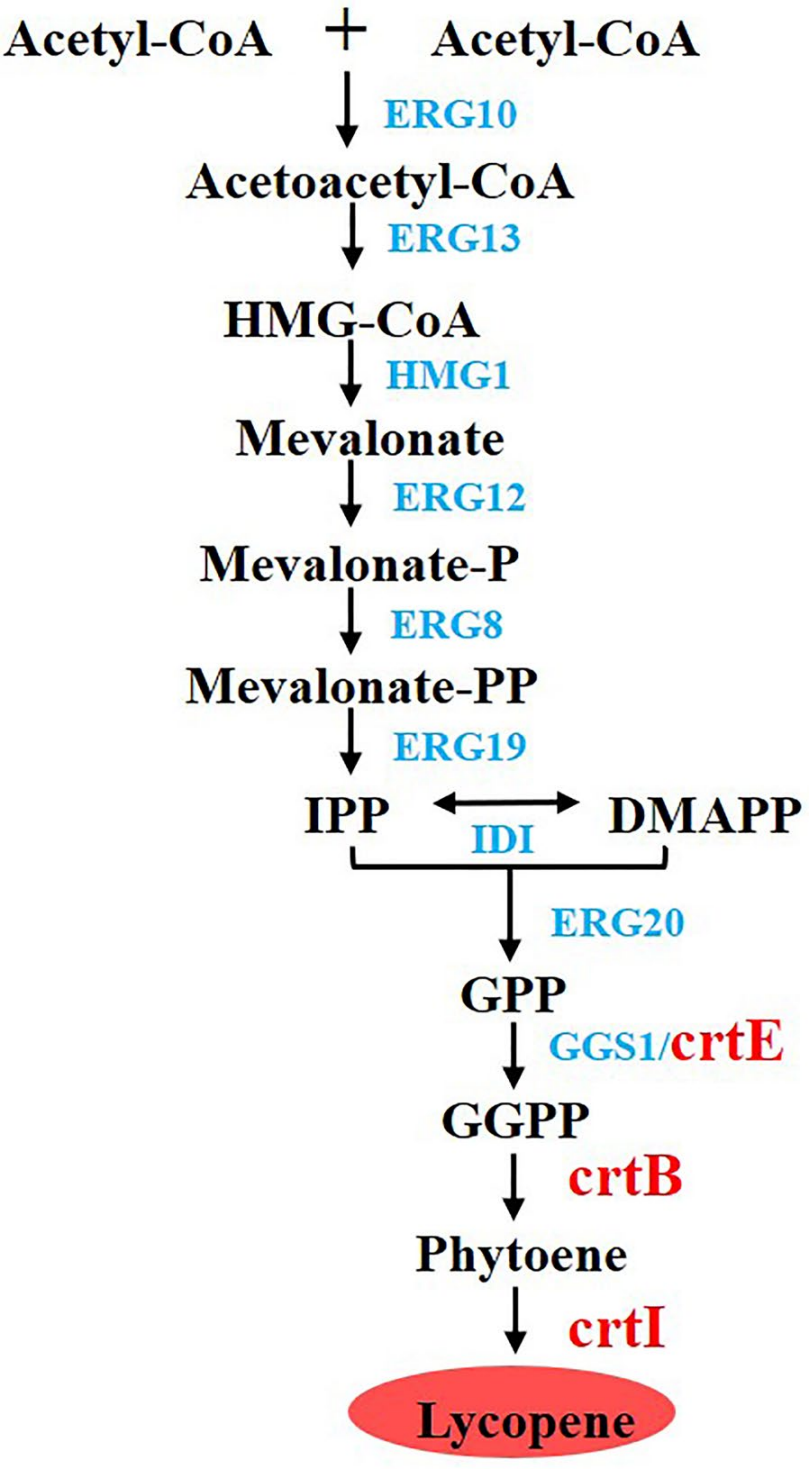
Metabolic pathway of lycopene synthesis in *Y. lipolytica*. In the upstream pathway, lycopene precursors are produced from acetyl-CoA via the MVA pathway; in the downstream pathway, *crt* genes achieve the heterogeneous biosynthesis of lycopene in *Y. lipolytica*. The blue-colored text indicates the endogenous enzymes of *Y. lipolytica*, whereas the text in red indicates the heterogenous enzymes. ERG10/AtoB: acetyl-CoA thiolase, ERG13: HMG-CoA synthase, HMG1/HMGR: 3-hydroxy-3-methyl glutaryl coenzyme A reductase, ERG12: mevalonate kinase, ERG8: phosphomevalonate kinase, ERG19: mevalonate diphosphate decarboxylase, IDI: IPP isomerase, ERG20: geranyl/farnesyl diphosphate synthase, GGS1/crtE: geranylgeranyl diphosphate synthase, crtB: phytoene synthase, crtI: phytoene desaturase, MVA: mevalonic acid, IPP: isopentenyl diphosphate, DMAPP: dimethylallyl diphosphate, GPP: geranyl pyrophosphate, FPP: farnesyl diphosphate, GGPP: geranylgeranyl diphosphate

*Y. lipolytica* does not contain a natural lycopene synthesis pathway; therefore, it requires the insertion of heterologous *crt* genes. The associated biosynthetic pathway has been well characterized. That is, lycopene is produced from the common terpenoid precursors isopentenyl diphosphate (IPP) and dimethylallyl diphosphate (DMAPP). In the upstream pathway, the MVA pathway utilizes acetyl-CoA to produce IPP, which is subsequently isomerized to generate DMAPP. In the downstream pathway, geranyl diphosphate synthase (ERG20) catalyzes the condensation of IPP and DMAPP to form farnesyl diphosphate (FPP), which can be condensed to geranylgeranyl diphosphate (GGPP) by GGPP synthase (GGS1/crtE) (Kampranis and Makris 2012; Vickers et al. 2017; Schempp et al. 2018). The condensation of two GGPP molecules by phytoene synthase (crtB) forms phytoene—a colorless compound. Lycopene is synthesized via the catalytic activity of phytoene desaturase (crtI) (Fig. 1) (Ma et al. 2015). Therefore, several strategies have been reported to enhance target production, including increasing acetyl-CoA precursor (Chen et al. 2013; Lian et al. 2014; Meadows et al. 2016), downregulating competitive pathways (Paddon et al. 2013; Peng et al. 2017; Gao et al. 2017), and lipid or membrane engineering(Ma et al. 2015; Hong et al. 2019; Wang et al. 2020).

As *Y. lipolytica* does not carry native plasmids, the available genetic tools are not as efficient as those for *S. cerevisiae*. Artificial vectors are not suitable for industrial applications because of their low copy numbers and high instability (Vernis et al. 2001). However, various biologically synthetic and genome editing tools have recently been developed for targeted, efficient, and multiple genetic modifications of *Y. lipolytica*, including the CRISPR/Cas9 system (Schwartz et al. 2016b; Holkenbrink et al. 2018), multiple copy gene integration with rDNA (Bulani et al. 2012; Gao et al. 2014; Lv et al. 2019) or zeta sites (Bordes et al. 2007), homology-independent randomly integration (Cui et al. 2019), and LoxP-mediated system (Zhang et al. 2021). *Y. lipolytica* contains more than 200 ribosomal DNA (rDNA) sites and 60 transformant copies, many of which are integrated into rDNA (Le Dall et al. 1994). Although homologous recombination (HR) is frequently used to achieve stable heterologous expression and genetic modification, these types of targeted modifications, require numerous templates with homology arms and have complicated operating procedures. Moreover, *Y. lipolytica* has very low HR efficiency, limiting the application of HR for targeted integration (Wagner and Alper 2016). Meanwhile, non-homologous end joining (NHEJ) is an alternative approach for integrating DNA into *Y. lipolytica*. NHEJ enables DNA fragments to be randomly integrated into different chromosomal sites, and has demonstrated remarkable efficacy in the construction of genomic libraries (Cui et al. 2019).

In this study, we improved lycopene production in *Y. lipolytica* using efficient gene editing tools and effective engineering strategies. A basic strain conducive for synthesizing tetraterpenes in *Y. lipolytica* was constructed by overexpressing the genes *HMG1* and *GGS1*. Additionally, the lycopene synthesis genes *crtE*, *crtB*, and *crtI* were expressed via rDNA-mediated multicopy random integration. To increase the flux from the acetyl-CoA precursor to synthesize more lycopene, the genes for MVA synthesis were optimally overexpressed via NHEJ-mediated multi-round iterative transformation.

In addition, the effects of lipid accumulation on cell growth and lycopene production were investigated. Ultimately, a strain capable of high lycopene yields was obtained by combining the MVA and lipid synthesis pathways. This study proposes an improved engineering strategy for *Y. lipolytica*-based lycopene production.

## Materials and methods

### Strains and culture conditions

*Y. lipolytica* strain ATCC MYA2613 (Po1f) was the chassis strain, and all *Y. lipolytica* engineered strains were cultivated at 30 °C in Yeast Extract Peptone Dextrose (YPD) medium (20 g/L glucose, 10 g/L yeast extract, 20 g/L tryptone, 25 mg/L chloromycetin). *E. coli* strain JM109 was used for plasmid construction and proliferation and was cultivated at 37 °C in LB medium (10 g/L NaCl, 10 g/L tryptone, 5 g/L yeast extract), with ampicillin (100 mg/L) or kanamycin (50 mg/L). YPD medium with 700 mg/L nourseothricin was used for antibiotic screening and 1 g/L 5-fluoroorotic acid (5-FOA) for recycling the URA3 selection marker (Schwartz et al. 2016a); the YNB medium (0.67% yeast nitrogen base without amino acids, 2% glucose) with specific nutritional markers (100 mg/L uracil or leucine) was used for transformant selection. The waste cooking oil, soybean oil, and oleic acid were added as the sole carbon source (1 mL into 50 mL YPD medium without glucose) or additives (500 μL into 50 mL YPD medium). Solid medium contained 1.5–2% agar based on corresponding medium. All strains constructed and used in this study are listed in Additional file 1: Table S1.

### Strains and plasmid construction

Endogenous genes were amplified from the genome of *Y. lipolytica* or plasmids constructed in our previous studies (Gao et al. 2018; Wei et al. 2021). Heterologous genes including lycopene synthesis genes *crtE*, *crtB* and *crtI* from *Lamprocystis purpurea* were optimized by Generay Biotech Co., Ltd. (Generay, Shanghai, China), and conducted in plasmid pINA1312, pINA1312 and pHR_F-1_hrGFP, respectively. Genes were with designed PCR primers that contained a ~ 20-bp homologous region to the plasmid backbone at both ends. The *HMG1* and *GGS1* genes were inserted into the *Pte*I and *Nhe*I sites of plasmids pHR_A1-2_hrGFP and pHR_A08_hrGFP to construct CRISPR/Cas9 system donor plasmids pHR_A1-2_HMG1 and pHR_A08_GGS1, respectively (Schwartz et al. 2016b). The *crtE*, *crtB* and *crtI* expression cassettes were sequentially inserted into the *EcoR*I site of pUC19-rDNA by enzyme digestion and seamless cloning to form the plasmid pUC19-rDNA-crtEBI, which were linearized with *Nde*I for further yeast transformation. Genes *AtoB* from *E. coli* and *HMGR* from *Bordetella petrii* were inserted into the plasmid pMD18T-HisG-NatR-HisG without the homologous arms, and were under the nourseothricin resistance marker; meanwhile, *AtoB* and endogenous *ERG13* were under the auxotrophic marker URA3. Related genes in MVA pathway, such as *IDI*, *ERG12*, *ERG13*, *ERG19* and *ERG20*, were constructed in pINA1269 and then digested with *BsrG*I. The *ACC1*, *DGA1*, *GPD1* expression cassettes were also inserted into pINA1269 individually or simultaneously, then digested with *Spe*I. Plasmid pINA1312-DGA1 and pINA1312-DGA1-GGS1 were linearized with *Not*I. The linearized plasmids and the constructed donor plasmids with gRNA plasmids were transformed to *Y. lipolytica* by the kit Frozen-EZ yeast transformation II. All primers used and plasmids constructed in this study are shown in Additional file 1: Tables S2 and S3.

### Genomic DNA library preparation and sequencing

DNA was extracted from the tissue using the CTAB method. Only high-quality DNA samples with OD260/280 ratios of 1.8–2.0 and OD260/230 ratios of 2.0 were used to construct the sequencing library.

Using Truseq Nano DNA HT Sample Prep Kit (Illumina USA) to generate the sequencing library. Initially, the genomic DNA sample was fragmented to a size of 350 bp through sonication. Then DNA fragments were end-polished, A-tailed, and ligated with the full-length adapter for Illumina sequencing, followed by further PCR amplification. After purifying the PCR products, the libraries were analyzed for size distribution using the Agilent 2100 Bioanalyzer and quantified by real-time PCR (3 nM). Finally, the paired-end DNA-seq sequencing library was sequenced with the Illumina NovaSeq system (Li et al. 2022; Sun et al. 2019).

### Shake-flask and 5-L fermentation

Before shake flask fermentation, the strain was lined on YPD solid medium, and one single colony was precultured in 2 mL of YPD medium. After cultivating overnight, the culture was vaccinated into 250-mL shake flasks containing 50 mL of YPD medium at an initial OD_600_ of 0.01 and cultivated for 4 days, and each shake flask cultivation was performed with two parallel replicate experiments. Additionally, 50 mL of seed culture from the 250-mL shake flask was transferred to a 5-L fermenter containing 2 L of 2 × YPD medium. The feeding medium was 500 g/L glycerol. The pH was controlled at 5.5 through 3 M HCl and NaOH, while the dissolved oxygen was controlled at 30% with an agitation cascade of 200–800 rpm, and an air flow of 1 vvm (Gao et al. 2020).

### Methods of extraction and analysis

The OD_600_ of samples was detected by UV–visible spectrophotometer. The dry cell weight (DCW) was calculated according to the weight increase after drying for 48 h in a 105 °C oven. That is, 2 mL of culture was centrifuged in a pre-weighed dry centrifuge tube (5 min, 12,000 rpm), and the cell pellets were washed twice with deionized water (Gao et al. 2018).

To extract lycopene, 50 μL of the fermentation culture was added into 200 μL of 3 M HCl, heated to 100 °C for 3 min, placed on ice for 4 min, and cooled immediately in an ice bath for 3 min. After centrifugation at 12,000 rmp for 5 min, the cell pellet was washed twice with water and resuspended in an acetone and DMSO mixture (1:1, v/v) (Chen et al. 2016; Zhang et al. 2019).

Lipid content was determined by collecting 20 mL of fermentation liquid in a 50-mL centrifuge tube, which was then centrifuged for 4 min and washed twice with water. Cell pellets were resuspended in 5 mL of 4 M HCl and mixed thoroughly, heated to 100 °C for 5 min, placed on ice for 5 min, and cooled immediately in an ice bath for 3 min; the steps were then repeated. Subsequently 20 mL of a methanol and chloroform solution (1:2, v/v) were added, mixed, and centrifuged (4 min, 6400 rpm). The lower liquid was removed and added to a pre-weighted glass tube. The weight was then determined after drying for 48 h in a 105 °C oven.

The SHIMADZU LC-20A high-performance liquid chromatography (HPLC) system equipped with an Agilent Zorbax C18 column (250 mm × 4.6 mm, 5 µm; Agilent Technologies Inc., Santa Clara, CA) and UV/VIS detection at 450 nm was used to quantify the lycopene content. The mobile phase comprised solution A (acetonitrile/water = 9:1, v/v) and B (methanol/isopropanol = 3:2, v/v) with a flow rate of 1 mL/min at 40 °C. The lycopene standard curves were prepared by running the same extraction process as the samples (Additional file 1: Figure S1).

## Results and discussion

### Heterogeneous synthesis of lycopene in *Y. lipolytica* via rDNA multicopy random integration

HMG-CoA reductase (HMG) is the primary rate-limiting enzyme in the MVA pathway; as such, it can increase the supply of FPP, a common compound that synthesizes numerous terpenoids (Bröker et al. 2018). GGS1 catalyzes FPP to the lycopene direct precursor GGPP. *GGS1* overexpression significantly enhances β-carotene production in different organisms (Verwaal et al. 2007; Matthäus et al. 2014). Furthermore, overexpression of *HMG1* and *GGS1* causes a ten-fold increase in β-carotene yield (Kildegaard et al. 2017). Similar to β-carotene, lycopene is also a typical tetraterpenoid, of which GGPP is a precursor. To enhance the heterologous synthesis of lycopene in *Y. lipolytica*, we added a copy of *HMG1* and *GGS1* using the CRISPR/Cas9 system to generate strain LZ2, which represents a basic *Y. lipolytica* strain for tetraterpene synthesis.

**Fig. 2:**
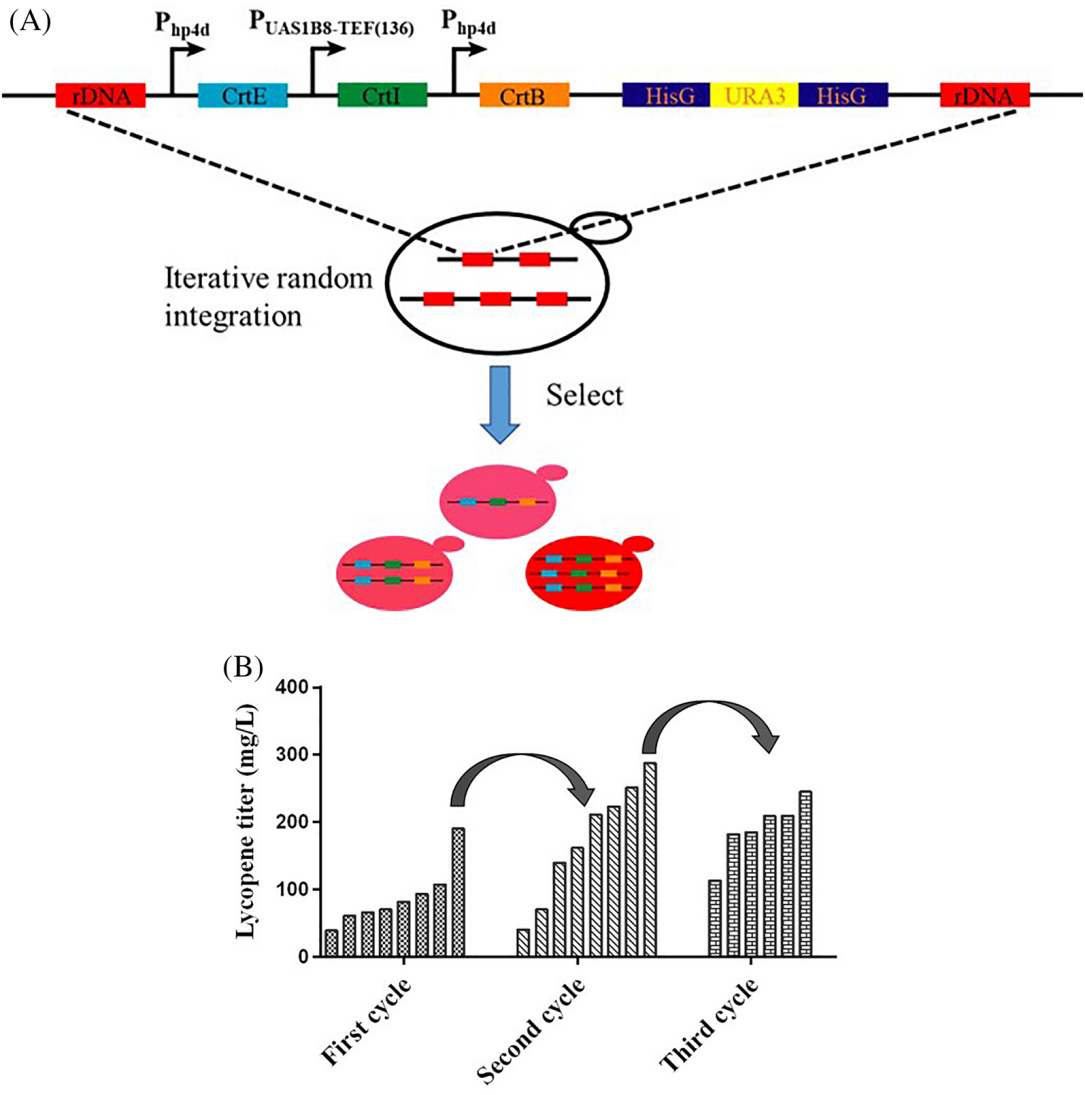
Screening lycopene-producing *Y. lipolytica* obtained via ribosomal DNA (rDNA) multicopy random integration. **A** Schematic diagram of rDNA multicopy random integration. **B** Lycopene production of different strains screened using multi-round random integration

The introduction of *crt* genes, including *crtE* (encoding GGPP synthase), *crtB* (encoding phytoene synthase), and *crtI* (encoding phytoene desaturase), is necessary to achieve heterogeneous biosynthesis of lycopene in *Y. lipolytica*. Recently, *crtE*, *crtB*, and *crtI* from *L. purpurea* achieved a final lycopene titer of 4.2 g/L, representing one of the highest reported *Y. lipolytica* yields (Luo et al. 2020). Xu et al. have demonstrated the efficacy of 26S rDNA iterative integration in enhancing the synthesis of flavonoids and hydroxylated flavonoids (Lv et al. 2019). Thus, *crtE* (GenBank: M38424.1), driven by the *hp4d* promoter, *crtB* (GenBank: M87280.1) driven by the *hp4d* promoter, and *crtI* (GenBank: M38423.1) driven by the *UAS1B8-TEF (136)* promoter *L. purpurea* (same as *P. agglomerans*) were randomly integrated into the rDNA sites of strain LZ2, such that the strains exhibited distinct phenotypes because to different genomic integration sites or gene copy numbers (Fig. 2A). The variants were screened based on their detectable lycopene titers, selection of the strain E12 that produced 190.3 mg/L lycopene was made. Iterative integration occurs at sufficiently high rates (> 80%) without disrupting the previous integration (Lv et al. 2019). As expected, in the second round of integration, strain E70 produced 288.2 mg/L lycopene, 51.4% higher than that of strain E12, which was obtained in the first round of transformation. However, a high yield was not obtained in the third round (Fig. 2B), suggesting that high integration rounds are not necessarily more conducive to increased lycopene production. This may be attributed to the overexpression of synthetic genes acting as a metabolic burden to host cells. Alternatively, no high-yield strains were screened because to the limited integration number.

During the modification process, uracil auxotrophic marker was used to identified the recombination strains in this study. Metabolic engineering significantly affected biomass and productivity of the engineered strain due to uracil auxotroph, which is consistent with previous report using the *Y. lipolytica* Po1f expression system (Blazeck et al. 2014). However, when the auxotroph was alleviated in the background strain E12 by blank plasmid pINA1312, the production of resulting strain did not reach the level that the strain E12 had (Additional file 1: Figure S2). We, therefore, opted to modify strain E12 using resistance screening in subsequent experiments to take advantage of its color-based screening process.

### *Optimized expression of key genes for MVA synthesis *via* NHEJ-mediated multi-round iterative transformation*

**Fig. 3:**
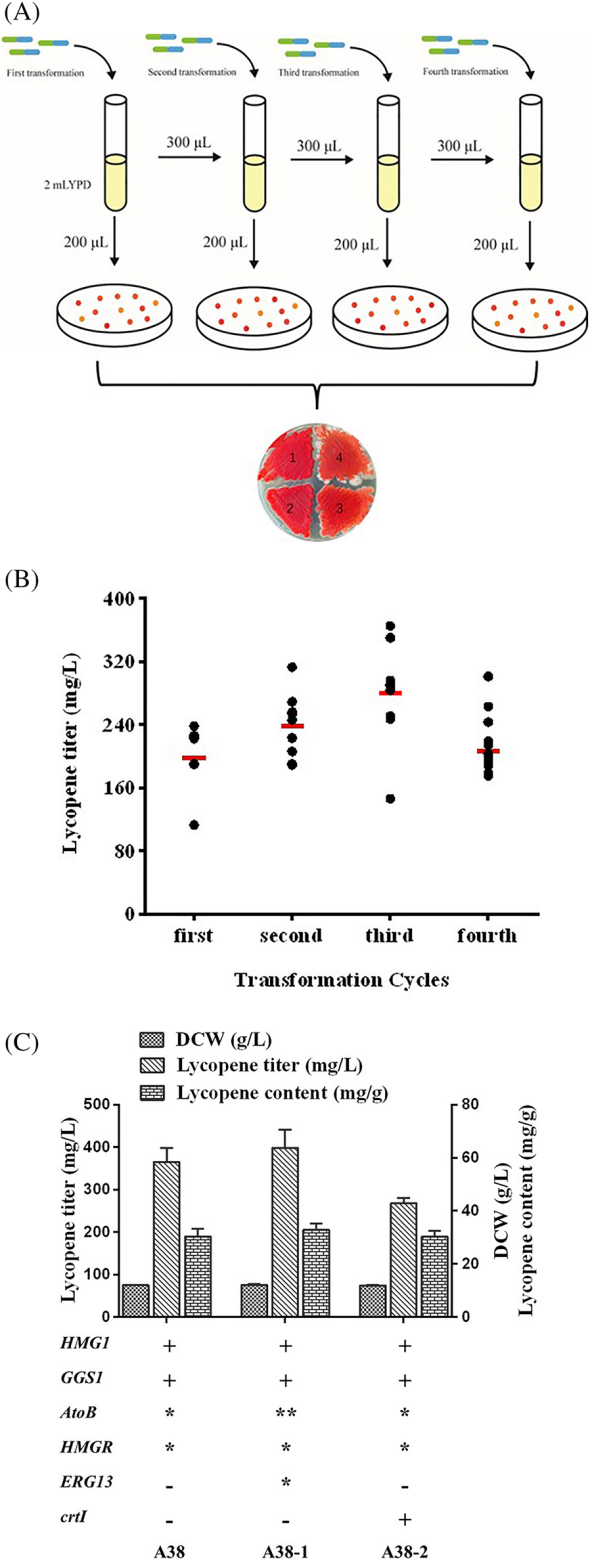
Optimized expression of *AtoB*, *HMGR,* and *ERG13* via non-homologous end joining (NHEJ)-mediated multi-round iterative transformation. **A** Schematic diagram of NHEJ-mediated multi-round iterative transformation, showing the color performance of each transformant on the plate; **B** lycopene production of transformants after expression of *AtoB* and *HMGR* via multi-round transformation. The red lines indicate the average yield of each round. **C** Lycopene production of different engineered strains; data are presented as the averages of two replicates with error bars representing standard deviations; “*” represents genes optimized via multi-round iterative transformation

A *Y. lipolytica* strain with codon-optimized *AtoB* from* E. coli* and NADH-dependent *HMGR* from *B. petrii,* as well as endogenous *ERG13* inserted into the genome via the NHEJ-mediated method, exhibits high MVA production (Liu et al. 2019). Moreover, to generate a large population of strains with diverse phenotypes, multi-round iterative transformation was conducted to generate strains with prominent phenotypes caused by transformation superposition (Bai et al. 2021). Thus, to increase the flux from the acetyl-CoA precursor to MVA, for the synthesis of lycopene, we attempted to overexpress *AtoB*, *HMGR*, and *ERG13* via NHEJ-mediated multi-round iterative transformation, conducted after the foreign DNA fragment without homology arms was transformed into strain E70 (Fig. 3A). Here, 200 µL of cell suspension, from the 500 µL transformation system, was spread on a YPD plate, whereas the remaining 300 µL was added to 2 mL of fresh YPD medium and cultured for 12–15 h in preparation for the next round of transformation. After four rounds of iterative transformations, strains with significant diversity were generated, and variants with distinct phenotypes were selected based on their darker colors. Therefore, NHEJ-mediated multi-round iterative transformations can achieve random and scattered genomic integration of multiple heterologous genes and obtain outstanding strains rapidly without removing inserted markers.

The simultaneous integration of these three long genes may have affected their efficiency; therefore, *AtoB* and *HMGR*, under the nourseothricin resistance marker, were expressed first. After four rounds of iterative transformations, approximately eight colonies from each transformant were selected for fermentation, and the transformant yield was analyzed in each round. Strain A38 was obtained in the third transformation round and had the highest lycopene titer (365.1 mg/L), which was 26.7% higher than that of strain E70 (Fig. 3B). Moreover, the average yield of the first three cycles exhibited an upward trend, however, a subsequent decrease was observed in the fourth cycle. These results indicated that NHEJ-mediated multi-round iterative transformations resulted in the optimal expression of key genes, perhaps because of the maximum self-selection ability of cells. The same method was then applied to integrate *AtoB* and *ERG13*, generating strain A38-1, which produced 398.5 mg/L lycopene, thus surpassing the yield of strain A38 (Fig. 3C). In summary, this iterative transformation method not only reduced the requirement for removing selection markers, but also optimized the expression of key genes and achieved the highest productivity.

A relatively high *crtI* transcript number is required for high lycopene production (Su et al. 2020). Among the three lycopene synthesis genes, enhanced expression of *crtI* proved most beneficial for lycopene production (Zhang et al. 2019). Thus, we sought to increase the *crtI* copy number, however, did not yield positive results with this strategy, increasing the *crtI* copy number might cause a higher metabolic burden for the engineered strain (Fig. 3C).

### Effective strategies for improving lycopene yield via the MVA pathway

To enhance lycopene production, we assessed the impact of up-regulating pertinent genes including *IDI*, *ERG12*, *ERG13*, *ERG19*, and *ERG20* within the MVA pathway. This may augment the flux from the acetyl-CoA precursor to MVA, thereby facilitating the biosynthesis of lycopene. As strain A38, with its high lycopene yield, was not screened in this set of experiments, the control strain A7, which also expressed *AtoB* and *HMGR* genes, and produced lycopene at a titer of 323.5 mg/L, was employed.

**Fig. 4:**
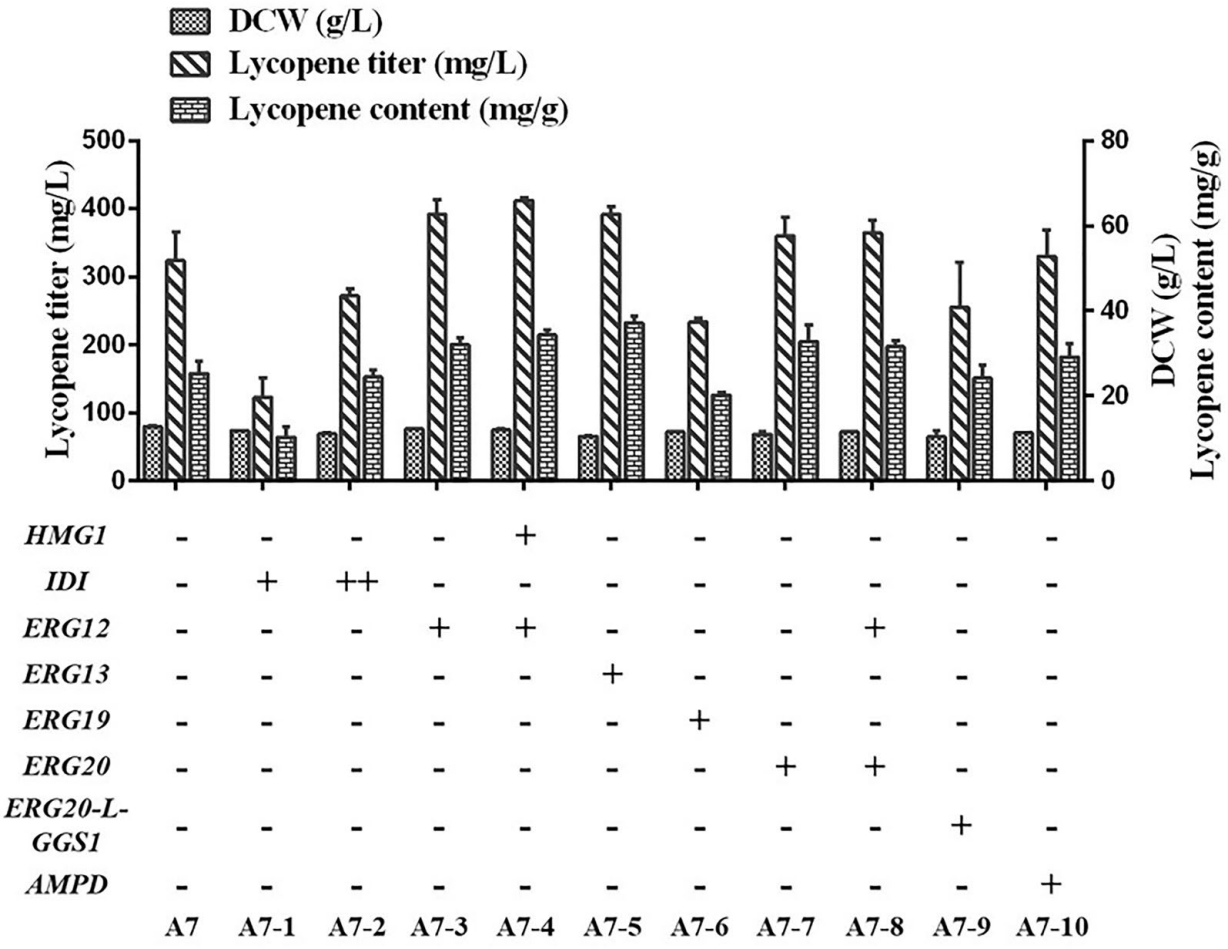
Effective strategies for improving lycopene yield via the MVA pathway. Lycopene production by different combinations of MVA pathway genes. Data are presented as the averages of two biological replicates with error bars representing standard deviations. HMG1: hydroxymethylglutaryl‐CoA reductase, IDI: isopentenyl diphosphate isomerase, ERG12: mevalonate kinase, ERG13: hydroxymethylglutaryl‐CoA synthase, ERG19: mevalonate diphosphate decarboxylase, ERG20: geranyl/farnesyl diphosphate synthase, AMPD: AMP deaminase, MVA: mevalonate

Overexpression of *ERG12*, *ERG13*, and *ERG20* increased lycopene production by 21.0%, 21.1%, and 11.3%, respectively (Fig. 4). In contrast, overexpression of *IDI* and *ERG19* reduced lycopene production*. HMG1* overexpression resulted in a lycopene yield by strain A7-4 (411.6 mg/L) that was slightly higher than that of strain A7-3 (391.3 mg/L), which was 27.2% higher than that of control strain A7. This may be attributed to an increase in *HMG1* copy number during the early stages; however, lycopene yield did not increase exponentially with increasing *HGM1* copy number.

Considering that GGS and ERG20 are adjacent enzymes, their coding genes were expressed by fusion combination to connect GGS with ERG20 using linker(G4S)2(GGTGGCGGTGGCAGCGGTGGCGGTGGCAGC). However, fusion expression was not conducive to lycopene production. Adenosine monophosphate deaminase (AMPD) can reportedly inhibit the activity of isocitrate dehydrogenase and, thus, generate a greater supply of acetyl-CoA precursor (Lu et al. 2019). Therefore, we postulated that overexpression of *AMPD* in *Y. lipolytica* can improve lipid production (Blazeck et al. 2014), which may contribute to increasing lycopene titers (Matthäus et al. 2014). However, *AMPD* overexpression did not significantly increase lycopene yield in our study.

Increasing metabolic flux flowing through the MVA pathway is a commonly used strategy to improve isoprenoid production. However, it is not necessary that each gene overexpression involving in MVA pathway would benefit for the desired end product (Cao et al. 2016). Overexpression of certain genes might cause a higher metabolic burden in the cells. In this study, we found that *ERG12* and *ERG13* expression positively affect lycopene yield, and the co-expression of *HMG1* and *ERG12* elicits optimal effects. Moreover, the resulting strain A7-4 exhibits a 27.2% higher lycopene yield (411.6 mg/L) than strain A7, suggesting that this strategy can be used to enhance lycopene production.

### Influence of lipid accumulation on lycopene production

**Fig. 5:**
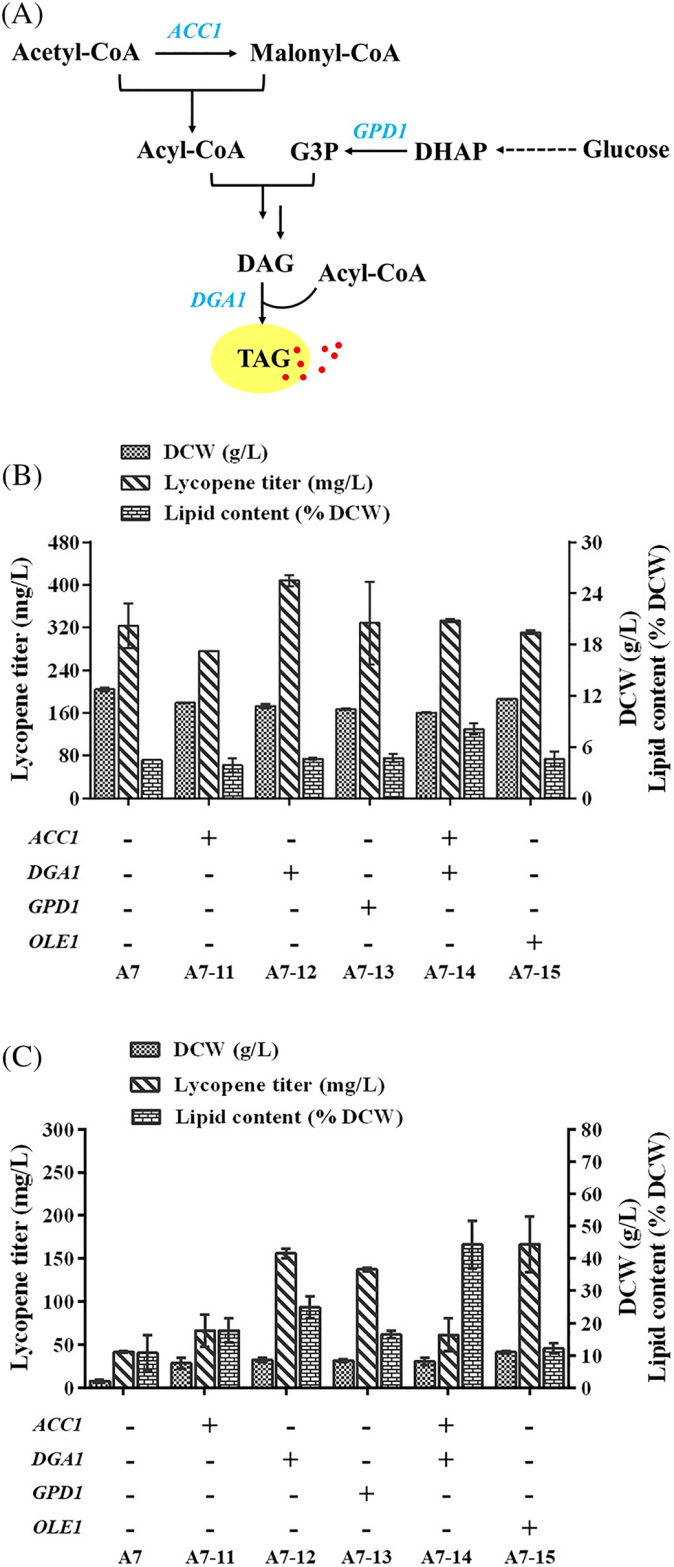
Influence of lipid accumulation on lycopene production. **A** TAG synthesis pathway. **B** Lycopene production of engineered strains in the YPD medium. **C** Lycopene production of engineered strains in the high carbon/nitrogen ratio medium

Lycopene belongs to a distinct group of lipophilic carotenoids and is nearly insoluble in ethanol, methanol, or water (Saini et al. 2015). Moreover, lipid accumulation is beneficial for the isolation and storage of lycopene (Ma et al. 2019; Luo et al. 2020). Therefore, theoretically, the higher the lipid content, the higher the lycopene yield. Acetyl-CoA carboxylase (ACC), diacylglycerol acyltransferase (DGA), and NAD-dependent G3P dehydrogenase (GPD) directly catalyze reactions for lipid synthesis or increase lipogenesis by removing glycerol head groups. That is, ACC is responsible for carboxylating acetyl-CoA to malonyl-CoA; DGA catalyzes the terminal step of triacylglycerol (TAG) formation; GPD is a key enzyme for glycerol synthesis (Fig. 5A). Previous reports have shown that overexpression of *ACC1*, *DGA1*, and *GPD1* leads to a high lipid-producing chassis, with approximately 16.5%, 28.7%, and 13.9% enhanced lipid content, respectively (Yang et al. 2019). Moreover, the simultaneous overexpression of *ACC1* and *DGA1* in *Y. lipolytica* created a strain with highly desirable phenotypes, including rapid cell growth and lipid overproduction (Qiao et al. 2015). Therefore, these three key enzymes were selected as targets to analyze the influence of lipid accumulation on lycopene production. Overexpression of fatty acid desaturase (OLE) increases the ratio of monounsaturated fatty acids to saturated fatty acids in cell membranes and improves the flexibility of the cell membrane, thus increasing lycopene yield (Fang et al. 2017; Hong et al. 2019); therefore, *OLE1*, encoding stearoyl-CoA 9-desaturase, was also overexpressed.

*DGA1* expression increased the lycopene titer by 26.2%, reaching 408.1 mg/L (Fig. 5B). Meanwhile, overexpression of *ACC1*, *DGA1*, and *GPD1* did not significantly impact lipid accumulation, save for co-expression of *ACC1* and *DGA1*, which resulted in few lipids accumulating. Considering that nitrogen-limited conditions are conducive to lipid synthesis, the YPD medium was replaced with a high carbon/nitrogen ratio medium (1.7 g/L YNB, 1.5 g/L yeast extract, 50 g/L glucose) (Yang et al. 2019). In this medium, the lipid content of all engineered strains increased with the co-expression of *ACC1* and *DGA1* resulting in lipid contents as high as 44.3% of the DCW. However, this increase in lipids had no impact on lycopene production in this study (Fig. 5C).

In addition, we investigated the effects of waste cooking oil, rapeseed oil, soybean oil, and oleic acid as the sole carbon and auxiliary carbon sources, on cell growth and lycopene production. Although these oils, particularly rapeseed oil, promoted cell growth, they, too, exerted no impact on lycopene production (Additional file 1: Figure S3). Therefore, lipid accumulation is associated with the culture medium composition, however, no direct relationship exists between lipid accumulation and lycopene production.

### Improvement in lycopene production by combining the MVA and lipid synthesis pathways

**Fig. 6:**
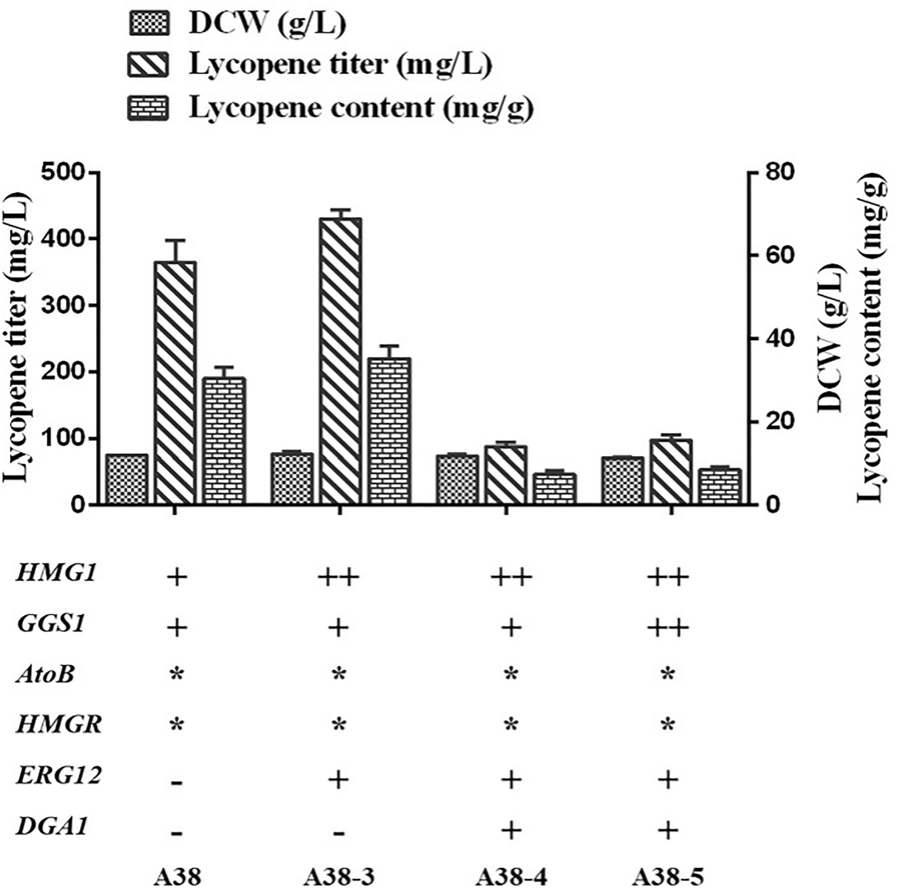
Combination of the mevalonate (MVA) and lipid synthesis pathways to improve lycopene production. Lycopene production of engineered strains via the better strategies in the MVA and lipid synthesis pathways. “*” represents genes optimized via multi-round iterative transformation

Following our exploration of the MVA pathway and lipid accumulation, the two strategies were combined to increase lycopene titer further. After *HMG1* and *ERG12* were overexpressed in strain A38, the lycopene yield increased by 17.9%, to 430.5 mg/L, which was the highest yield obtained in this study. However, when *DGA1* was overexpressed in strain A38-3, lycopene production decreased. Attempts to increase the *GGS1* copy number also failed to improve lycopene production (Fig. 6). This may have been caused by *DGA1* competing with the acetyl-CoA required by the MVA pathway. Therefore, the optimal allocation of acetyl-CoA must be achieved between the MVA and lipid synthesis pathways. It is also possible that either the repeated application of the random integration method impacted the genome structure, or different expression vectors might have led to different effects. In order to determine the insertion sites and gene copies of the fragments of *CrtE*/*CrtB*/*CrtI* and *AtoB*/*HMGR*, we applied whole genome sequencing for the best lycopene-producing strain A38-3. As shown in Additional file 1: Figure S5, the possible insertion sites of *CrtE*/*CrtB*/*CrtI* and *AtoB*/*HMGR* expression cassette were distributed on chromosome C and F. And the copy number of *CrtE*/*CrtB*/*CrtI* and *AtoB*/*HMGR* was 2 and 1, respectively. The fewer integration numbers of *CrtE*/*CrtB*/*CrtI* and *AtoB*/*HMGR* expression cassette in this study might lead to lower lycopene production than previous report (Bai et al. 2021).

### Optimization of fed-batch fermentation to increase lycopene production

**Fig. 7:**
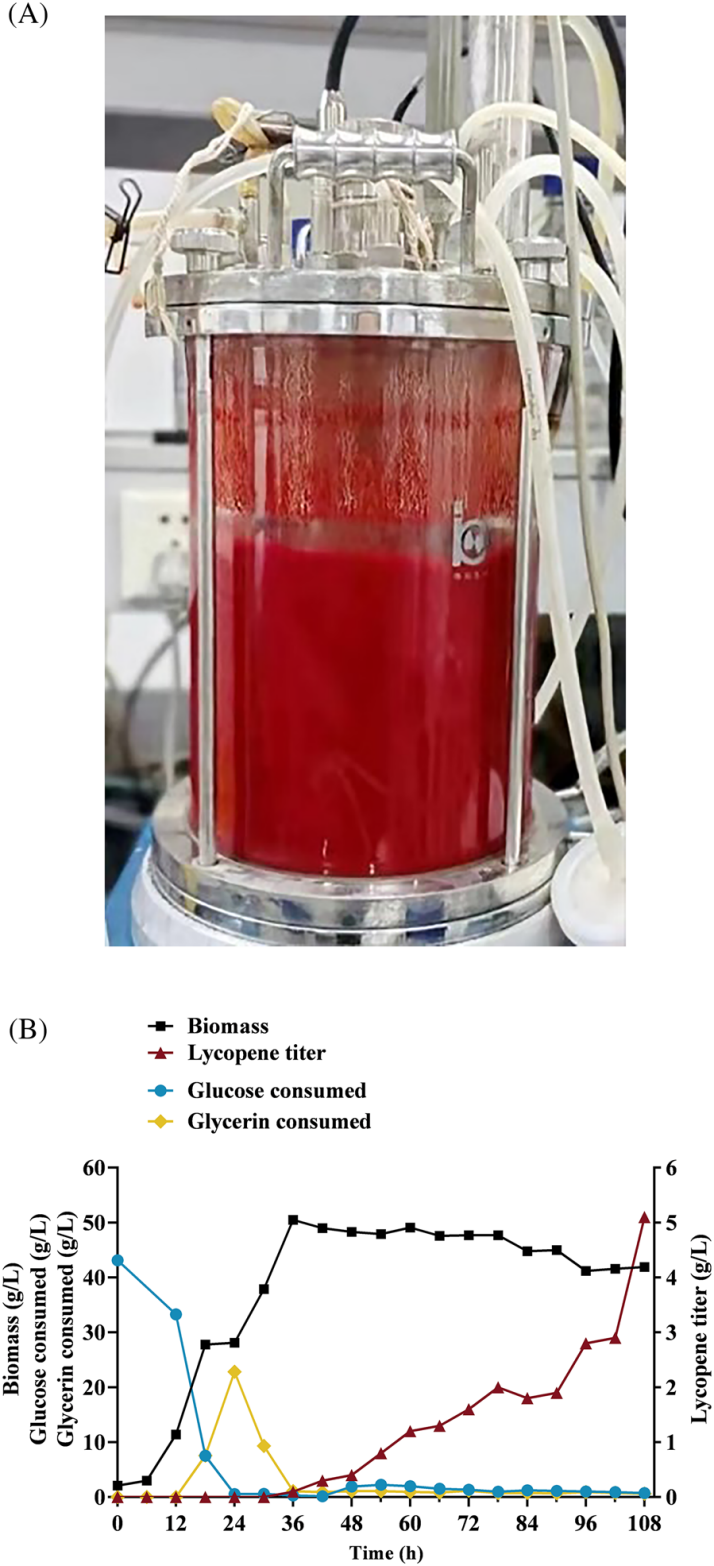
Optimization of fed-batch fermentation of A38-3. **A** The color of strain A38-3 after 108 h in a 5-L bioreactor. **B** Batch fermentation results of strain A38-3

Glycerol is an ideal substrate for *Y. lipolytica*. Indeed, cells perform better in glycerol-containing media than in glucose-containing media (Czajka et al. 2018; Jacobsen et al. 2020). Some reports have shown that glycerol was superior to glucose as a carbon source in the production of isoprenoids such as limonene, farnesene and ꞵ-carotene by *Y. lipolytica* (Liu et al. 2020; Yao et al. 2020). Glycerol can be catabolized into various organic acids, which might facilitate lycopene production. To evaluate the potential effects of the addition of organic acid on lycopene production, citrate, pyruvate, acetate, and malate (concentrations varying from 0 to 500 mg/L) were supplemented in the YPD medium. The result showed that the culture by malate supplementation was the most effective for increasing lycopene production (Additional file 1: Figure S4). To further improve lycopene production, Strain A38-3, with the highest lycopene yield in this study, was evaluated in 5-L fed-batch cultivations with glycerol as the carbon source. The fermentation medium was a 2 × YPD medium with an initial glucose concentration of 40 g/L. Following glucose consumption, 500 g/L of glycerol was used for feeding at a rate of 8 mL/h. The color of strain A38-3 after 108 h, and the biomass and lycopene titer in the fermentation process, are indicated in Fig. 7A and B. The cells underwent rapid growth in the first 36 h (Fig. 7B), and entered a stable phase, ultimately resulting in a 50 g/L biomass yield. Although cells did not accumulate lycopene in the first 36 h, the yield increased gradually thereafter, peaking at 5.1 g/L by 108 h. Finally, strain A38-3 produced 121 mg/g DCW of lycopene in a 5-L fed-batch fermentation system. The growth and production periods of the strain are separated; that is, glucose was rapidly and nearly completely consumed by 24 h. Meanwhile, although glycerol was introduced at the 12 h point, it was consumed only after glucose had been completely consumed, likely because of existing glucose repression. Subsequently, the glycerol concentration remained low after the cells entered the stable phase.

*Y. lipolytica* has been used for lycopene production. The highest lycopene yield reported in 2022 was 17.6 g/L (313 mg/g) in a 3 L fermenter with the feeding medium optimized based on the C/N ratio (Ma et al. 2022). In addition, a *Y. lipolytica* strain engineered in 2020 produced 1.6 g/L lycopene in a shaking flask and 4.2 g/L lycopene in a 3-L fermenter. The strain was engineered by constructing an exogenous IUP pathway to enhance the IPP and DMAPP precursors in the natural MVA pathway; the growth medium was enhanced with dodecane and isoprenol (Luo et al. 2020). Although the lycopene yield obtained in the current study was not higher than the highest yield previously reported, the medium used in this study was not enhanced using other substances (Table 1).

**Table 1:** The advances of lycopene production by *Y. lipolytica*

Substrate	Method	Cultivation mode	Titer/yield	References
Glucose	Adjusted of the copy number of three heterologous lycopene biosynthesis genes (*crtE*, *crtB* and *crtI*) and overexpressed of AMP deaminase coding gene (AMPD)	Fed-batch	46–60 mg/g	Zhang et al. (2019)
Mixed substrate (glucose and fatty acid et al.)	Synthesized of carotenoid precursors IPP and DMAPP through the IUP	Fed-batch	4.2 g/L	Luo et al. (2020)
Glucose	Released the substrate inhibition of lycopene cyclase	Fed-batch	17.6 g/L	Ma et al. (2022)
Glucose	Overexpressed of endogenous HMGS from *Y. lipolytica* and HMGR from *Bordetella petrii*	Fed-batch	1.96 g/L	Cui et al. (2019)
Glucose	Deleted of POX1-POX6 and GUT2, expressed of P*acrtB*, P*acrtI*, *crtE* and *hmgr*	Fed-batch	16 mg/g DCW	Gatter et al. (2016)
Glycerol	Employed different methods to improve the key genes overexpression including *crtE*, *crtB*, *crtI*, *AtoB*, *HMGR*, *ERG12*	Fed-batch	5.1 g/L	This study

## Conclusion

In this study, we systematically engineered *Y. lipolytica* for high-yield lycopene production. A series of strategies were applied to improve heterologous lycopene biosynthesis, including optimizing key genes in the MVA and lipid synthesis pathways. Different methods were employed to improve the gene editing efficiency, including the CRISPR/Cas9 system, rDNA multicopy random integration, and NHEJ-mediated multi-round iterative transformation. These strategies and tools resulted in a lycopene titer of 5.1 g/L in a 5-L fed-batch fermentation system. Our results may provide a reference for increasing the production of other tetraterpenoids in *Y. lipolytica*.

The methods of rDNA multicopy random integration and NHEJ-mediated multi-round iterative transformation improved the efficiency of gene editing and established a multi-strain library. Combining these tools with a high-throughput screening strategy may facilitate more effective screening of high-yield strains, and realize the full potential for synthesizing of high-value compounds. Meanwhile, the combination of the MVA and lipid synthesis pathways decreased lycopene production. Therefore, optimizing the allocation of acetyl-CoA between the MVA and lipid synthesis pathways is essential; moreover, the application of promoter engineering and organelle localization strategies may further improve lycopene production.

### Supplementary Information

**Additional file 1: Table S1.** Strains used in this study. **Table S2.** Plasmids used in this study. **Table S3.** Primers used in this study. **Figure S1.** Lycopene standard curve. **Figure S2.** Comparison of lycopene production before and after recovering the selection marker URA3. **Figure S3.** Effects of different oil substances on strain growth and lycopene yield. **Figure S4.** Effects of organic acids on lycopene yield. **Figure S5.** Whole genome sequencing demonstrated random and scattered insertions of *CrtE*/*CrtB*/*CrtI* and *AtoB*/*HMGR* by rDNA and NHEJ-mediated multi-round iterative transformation.

## Data Availability

All data generated or analyzed during this study are included in this article.

## References

[CR1] Aghajanpour M, Nazer MR, Obeidavi Z (2017). Functional foods and their role in cancer prevention and health promotion: a comprehensive review. Am J Cancer Res.

[CR2] Araya-Garay JM, Feijoo-Siota L, Rosa-dos-Santos F (2012). Construction of new *Pichia pastoris* X-33 strains for production of lycopene and β-carotene. Appl Microbiol Biotechnol.

[CR3] Bai Q, Cheng S, Zhang J (2021). Establishment of genomic library technology mediated by non-homologous end joining mechanism in *Yarrowia lipolytica*. Sci China Life Sci.

[CR4] Blazeck J, Hill A, Liu L (2014). Harnessing *Yarrowia lipolytica* lipogenesis to create a platform for lipid and biofuel production. Nat Commun.

[CR5] Bordes F, Fudalej F, Dossat V (2007). A new recombinant protein expression system for high-throughput screening in the yeast *Yarrowia lipolytica*. J Microbiol Methods.

[CR6] Bröker JN, Müller B, van Deenen N (2018). Upregulating the mevalonate pathway and repressing sterol synthesis in *Saccharomyces cerevisiae* enhances the production of triterpenes. Appl Microbiol Biotechnol.

[CR7] Bulani SI, Moleleki L, Albertyn J, Moleleki N (2012). Development of a novel rDNA based plasmid for enhanced cell surface display on *Yarrowia lipolytica*. AMB Express.

[CR8] Cao X, Lv YB, Chen J, Imanaka T, Wei LJ, Hua Q (2016). Metabolic engineering of oleaginous yeast *Yarrowia lipolytica* for limonene overproduction. Biotechnol Biofuels.

[CR9] Chen Y, Daviet L, Schalk M (2013). Establishing a platform cell factory through engineering of yeast acetyl-CoA metabolism. Metab Eng.

[CR10] Chen Y, Xiao W, Wang Y (2016). Lycopene overproduction in *Saccharomyces cerevisiae* through combining pathway engineering with host engineering. Microb Cell Fact.

[CR11] Coussement P, Bauwens D, Maertens J, De Mey M (2017). Direct combinatorial pathway optimization. ACS Synth Biol.

[CR12] Cui Z, Jiang X, Zheng H (2019). Homology-independent genome integration enables rapid library construction for enzyme expression and pathway optimization in *Yarrowia lipolytica*. Biotechnol Bioeng.

[CR13] Czajka JJ, Nathenson JA, Benites VT (2018). Engineering the oleaginous yeast *Yarrowia lipolytica* to produce the aroma compound β-ionone. Microb Cell Fact.

[CR14] Fang Z, Chen Z, Wang S (2017). Overexpression of OLE1 enhances cytoplasmic membrane stability and confers resistance to cadmium in *Saccharomyces cerevisiae*. Appl Environ Microbiol.

[CR15] Gao S, Han L, Zhu L (2014). One-step integration of multiple genes into the oleaginous yeast *Yarrowia lipolytica*. Biotechnol Lett.

[CR16] Gao S, Tong Y, Zhu L (2017). Iterative integration of multiple-copy pathway genes in *Yarrowia lipolytica* for heterologous β-carotene production. Metab Eng.

[CR17] Gao Q, Cao X, Huang Y-Y (2018). Overproduction of fatty acid ethyl esters by the oleaginous yeast *Yarrowia lipolytica* through metabolic engineering and process optimization. ACS Synth Biol.

[CR18] Gao Q, Yang J-L, Zhao X-R (2020). *Yarrowia lipolytica* as a metabolic engineering platform for the production of very-long-chain wax esters. J Agric Food Chem.

[CR19] Gatter M, Ottlik S, Kovesi Z, Bauer B, Matthaus F, Barth G (2016). Three alcohol dehydrogenase genes and one acetyl-CoA synthetase gene are responsible for ethanol utilization in *Yarrowia lipolytica*. Fungal Genet Biol.

[CR20] Holkenbrink C, Dam MI, Kildegaard KR (2018). EasyCloneYALI: CRISPR/Cas9-based synthetic toolbox for engineering of the yeast *Yarrowia lipolytica*. Biotechnol J.

[CR21] Hong J, Park S-H, Kim S (2019). Efficient production of lycopene in *Saccharomyces cerevisiae* by enzyme engineering and increasing membrane flexibility and NAPDH production. Appl Microbiol Biotechnol.

[CR22] Jacobsen IH, Ledesma-Amaro R, Martinez JL (2020). Recombinant β-carotene production by *Yarrowia lipolytica*-assessing the potential of micro-scale fermentation analysis in cell factory design and bioreaction optimization. Front Bioeng Biotechnol.

[CR23] Jhou B-Y, Song T-Y, Lee I (2017). Lycopene inhibits metastasis of human liver adenocarcinoma SK-Hep-1 cells by downregulation of NADPH oxidase 4 protein expression. J Agric Food Chem.

[CR24] Kampranis SC, Makris AM (2012). Developing a yeast cell factory for the production of terpenoids. Comput Struct Biotechnol J.

[CR25] Kildegaard K, Adiego-Pérez B, Belda D (2017). Engineering of *Yarrowia lipolytica* for production of astaxanthin. Synth Syst Biotechnol.

[CR26] Le Dall MT, Nicaud JM, Gaillardin C (1994). Multiple-copy integration in the yeast *Yarrowia lipolytica*. Curr Genet.

[CR27] Li S, Wang C, You C, Zhou X, Zhou H (2022). T-LOC: a comprehensive tool to localize and characterize T-DNA integration sites. Plant Physiol.

[CR28] Lian J, Si T, Nair NU, Zhao H (2014). Design and construction of acetyl-CoA overproducing *Saccharomyces cerevisiae* strains. Metab Eng.

[CR29] Liu Y, Jiang X, Cui Z (2019). Engineering the oleaginous yeast *Yarrowia lipolytica* for production of α-farnesene. Biotechnol Biofuels.

[CR30] Lu X, Liu Y, Yang Y (2019). Constructing a synthetic pathway for acetyl-coenzyme A from one-carbon through enzyme design. Nat Commun.

[CR31] Liu SC, Liu Z, Wei LJ, Hua Q (2020) Pathway engineering and medium optimization for alpha-farnesene biosynthesis in oleaginous yeast *Yarrowia lipolytica*. J biotechnol 319:74–81. 10.1016/j.jbiotec.2020.06.005 10.1016/j.jbiotec.2020.06.005 32533992

[CR32] Luo Z, Liu N, Lazar Z (2020). Enhancing isoprenoid synthesis in *Yarrowia lipolytica* by expressing the isopentenol utilization pathway and modulating intracellular hydrophobicity. Metab Eng.

[CR33] Lv Y, Edwards H, Zhou J, Xu P (2019). Combining 26s rDNA and the Cre-loxP system for iterative gene integration and efficient marker curation in *Yarrowia lipolytica*. ACS Synth Biol.

[CR34] Ma T, Deng Z, Liu T (2015). Microbial production strategies and applications of lycopene and other terpenoids. World J Microbiol Biotechnol.

[CR35] Ma T, Shi B, Ye Z (2019). Lipid engineering combined with systematic metabolic engineering of *Saccharomyces cerevisiae* for high-yield production of lycopene. Metab Eng.

[CR36] Ma Y, Li W, Mai J (2021). Engineering *Yarrowia lipolytica* for sustainable production of the chamomile sesquiterpene (−)-α-bisabolol. Green Chem.

[CR37] Ma Y, Liu N, Greisen P (2022). Removal of lycopene substrate inhibition enables high carotenoid productivity in *Yarrowia lipolytica*. Nat Commun.

[CR38] Matthäus F, Ketelhot M, Gatter M, Barth G (2014). Production of lycopene in the non-carotenoid-producing yeast *Yarrowia lipolytica*. Appl Environ Microbiol.

[CR39] Meadows A, Hawkins K, Tsegaye Y (2016). Rewriting yeast central carbon metabolism for industrial isoprenoid production. Nature.

[CR40] Michael McClain R, Bausch J (2003). Summary of safety studies conducted with synthetic lycopene. Regul Toxicol Pharmacol.

[CR41] Mirończuk AM, Rzechonek DA, Biegalska A (2016). A novel strain of *Yarrowia lipolytica* as a platform for value-added product synthesis from glycerol. Biotechnol Biofuels.

[CR42] Nicaud J-M (2012). Yarrowia lipolytica. Yeast.

[CR43] Nogué VSI, Black BA, Kruger JS (2018). Integrated diesel production from lignocellulosic sugars via oleaginous yeast. Green Chem.

[CR44] Paddon CJ, Westfall PJ, Pitera DJ (2013). High-level semi-synthetic production of the potent antimalarial artemisinin. Nature.

[CR45] Peng B, Plan MR, Chrysanthopoulos P (2017). A squalene synthase protein degradation method for improved sesquiterpene production in *Saccharomyces cerevisiae*. Metab Eng.

[CR46] Qiao K, Imam Abidi SH, Liu H (2015). Engineering lipid overproduction in the oleaginous yeast *Yarrowia lipolytica*. Metab Eng.

[CR47] Saini RK, Nile SH, Park SW (2015). Carotenoids from fruits and vegetables: chemistry, analysis, occurrence, bioavailability and biological activities. Food Res Int.

[CR48] Schempp FM, Drummond L, Buchhaupt M, Schrader J (2018). Microbial cell factories for the production of terpenoid flavor and fragrance compounds. J Agric Food Chem.

[CR49] Schwartz C, Shabbir Hussain M, Frogue K (2016). Standardized markerless gene integration for pathway engineering in *Yarrowia lipolytica*. ACS Synth Biol.

[CR50] Schwartz CM, Hussain MS, Blenner M, Wheeldon I (2016). Synthetic RNA polymerase III promoters facilitate high-efficiency CRISPR–Cas9-mediated genome editing in *Yarrowia lipolytica*. ACS Synth Biol.

[CR51] Shi J, Le Maguer M (2000). Lycopene in tomatoes: chemical and physical properties affected by food processing. Crit Rev Food Sci Nutr.

[CR52] Shi B, Ma T, Ye Z (2019). Systematic metabolic engineering of *Saccharomyces cerevisiae* for lycopene overproduction. J Agric Food Chem.

[CR53] Su B, Song D, Yang F, Zhu H (2020). Engineering a growth-phase-dependent biosynthetic pathway for carotenoid production in *Saccharomyces cerevisiae*. J Ind Microbiol Biotechnol.

[CR54] Sun L, Ge Y, Sparks JA, Robinson ZT, Cheng X, Wen J, Blancaflor EB (2019). TDNAscan: A Software to Identify Complete and Truncated T-DNA Insertions. Front Genet.

[CR55] Vernis L, Poljak L, Chasles M (2001). Only centromeres can supply the partition system required for ARS function in the yeast *Yarrowia lipolytica*. J Mol Biol.

[CR56] Verwaal R, Wang J, Meijnen J-P (2007). High-level production of β-carotene in *Saccharomyces cerevisiae* by successive transformation with carotenogenic genes from *Xanthophyllomyces dendrorhous*. Appl Environ Microbiol.

[CR57] Vickers CE, Williams TC, Peng B, Cherry J (2017). Recent advances in synthetic biology for engineering isoprenoid production in yeast. Curr Opin Chem Biol.

[CR58] Wagner JM, Alper HS (2016). Synthetic biology and molecular genetics in non-conventional yeasts: current tools and future advances. Fungal Genet Biol.

[CR59] Wang Z, Li X, Yu C (2020). Continuous self-cycling fermentation leads to economical lycopene production by *Saccharomyces cerevisiae*. Front Bioeng Biotechnol.

[CR60] Wei L-J, Zhong Y-T, Nie M-Y (2021). Biosynthesis of α-pinene by genetically engineered *Yarrowia lipolytica* from low-cost renewable feedstocks. J Agric Food Chem.

[CR61] Wu T, Ye L, Zhao D (2017). Membrane engineering—a novel strategy to enhance the production and accumulation of β-carotene in *Escherichia coli*. Metab Eng.

[CR62] Yang K, Qiao Y, Li F (2019). Subcellular engineering of lipase dependent pathways directed towards lipid related organelles for highly effectively compartmentalized biosynthesis of triacylglycerol derived products in *Yarrowia lipolytica*. Metab Eng.

[CR63] Yao F, Liu SC, Wang DN, Liu ZJ, Hua Q, Wei LJ (2020). Engineering oleaginous yeast *Yarrowia lipolytica* for enhanced limonene production from xylose and lignocellulosic hydrolysate. FEMS Yeast Res.

[CR64] Zhang X-K, Nie M-Y, Chen J (2019). Multicopy integrants of crt genes and co-expression of AMP deaminase improve lycopene production in *Yarrowia lipolytica*. J Biotechnol.

[CR65] Zhang Y, Chiu T-Y, Zhang J-T (2021). Systematical engineering of synthetic yeast for enhanced production of lycopene. Bioengineering (basel).

