# Supplementary material for: Iterative gene integration mediated by 26S rDNA and non-homologous end joining for the efficient production of lycopene in Yarrowia lipolytica
Source: Bioresour Bioprocess. 2023 Nov 24;10(1):83. doi: 10.1186/s40643-023-00697-6 (PMC10992032; doi:10.1186/s40643-023-00697-6)
Supplement: Supplementary file 1 — Additional file 1: Table S1. Strains used in this study. Table S2. Plasmids used in this study. Table S3. Primers used in this study. Figure S1. Lycopene standard curve. Figure S2. Comparison of lycopene production before and after recovering the selection marker URA3. Figure S3. Effects of different oil substances on strain growth and lycopene yield. Figure S4. Effects of organic acids on lycopene yield. Figure S5. Whole genome sequencing demonstrated random and scattered insertions of CrtE/CrtB/CrtI and AtoB/HMGR by rDNA and NHEJ-mediated multi-round iterative transformation. [file 40643_2023_697_MOESM1_ESM.docx]

Research Article

**Iterative gene integration mediated by 26S rDNA and non-homologous end joining for the efficient production of lycopene in** ***Yarrowia lipolytica***Zhen Luo ^1#^ ∙ Jiang-Ting Shi ^1#^ ∙ Xin-Liang Chen ^1^ ∙ Jun Chen ^1^ ∙ Feng Liu ^1^ ∙ Liu-Jing Wei ^1^* ∙ Qiang Hua ^1,2^*

Table S1 Strains used in this study

| Strains | Descriptions | Source |
| --- | --- | --- |
| *E. coli* JM109 | recA1, endA1, gyrA96, thi, hsdR17, supE44, relA1, Δ (lac-proAB)/F [traD36, proab^+^, lacI^q^, lacZΔM15] | Invitrogen |
| *Y. lipolytica* Po1f | MatA, leu2-270, ura3-302, xpr2-322, axp1-2 | ^[1]^ |
| LZ1 | Po1f harboring UAS1B8-TEF (136)-HMG1-CYC:: A1-2 | This study |
| LZ2 | LZ1 harboring UAS1B8-TEF (136)-GGS1-CYC::A08 | This study |
| E12 | LZ2 harboring pUC19-rDNA-crtE-crtI-crtB | This study |
| E70 | E12 harboring pUC19-rDNA-crtE-crtI-crtB | This study |
| A7 | E70 harboring NHEJ-HMGR-AtoB | This study |
| A38 | E70 harboring NHEJ-HMGR-AtoB | This study |
| A38-1 | A38 harboring NHEJ-AtoB-ERG13 | This study |
| A38-2 | A38 harboring NHEJ-crtI | This study |
| A7-1 | A7 harboring pINA1269-IDI | This study |
| A7-2 | A7 harboring pINA1269-IDI-IDI | This study |
| A7-3 | A7 harboring pINA1269-ERG12 | This study |
| A7-4 | A7 harboring pINA1269-HMG1-ERG12 | This study |
| A7-5 | A7 harboring pINA1269-ERG13 | This study |
| A7-6 | A7 harboring pINA1269-ERG19 | This study |
| A7-7 | A7 harboring pINA1269-ERG20 | This study |
| A7-8 | A7 harboring pINA1269- ERG20-ERG12 | This study |
| A7-9 | A7 harboring pINA1269-ERG20-L-GGS1 | This study |
| A7-10 | A7 harboring pINA1269-AMPD | This study |
| A7-11 | A7 harboring pINA1269-ACC1 | This study |
| A7-12 | A7 harboring pINA1269-DGA1 | This study |
| A7-13 | A7 harboring pINA1269-GPD1 | This study |
| A7-15 | A7 harboring pINA1269-ACC1-DGA1 | This study |
| A7-15 | A7 harboring pINA1269-OLE1 | This study |
| A38-3 | A38 harboring pINA1269-HMG1-ERG12 | This study |
| A38-4 | A38-3 harboring pINA1312-DGA1 | This study |
| A38-5 | A38-4 harboring pINA1312-DGA1-GGS1 | This study |

Table S2 Plasmids used in this study

| Plasmids | Descriptions | Source |
| --- | --- | --- |
| pHR_A1-2_hrGFP | HR donor to integrate *hrGFP* into A1-2 locus | ^[2]^ |
| pHR_A08_hrGFP | HR donor to integrate *hrGFP* into A08 locus | ^[2]^ |
| pHR_A1-2_HMG1 | HR donor to integrate *HMG1* into A1-2 locus | This study |
| pHR_A08_GGS1 | HR donor to integrate *GGS1* into A08 locus | This study |
| pCRISPRyl_A1-2 | pCRISPRyl with A1-2 targeting sgRNA | ^[2]^ |
| pCRISPRyl_A08 | pCRISPRyl with A08 targeting sgRNA | ^[2]^ |
| pINA1312 | *Y. lipolytica* integrative plasmid with P_hp4d_ and T_XPR2_，Kan^R^ | ^[3]^ |
| pINA1312-crtE | pINA1312 vector containing *crtE* gene | This study |
| pINA1312-crtB | pINA1312 vector containing *crtB* gene | This study |
| pINA1312-crtI | pINA1312 vector containing *crtI* gene | This study |
| pHR_F-1_crtI | HR donor to integrate P_hp4d_-*crtI*-T_XPR2_ into F-1locus | This study |
| pUC19-rDNA-hisG-URA3-hisG | HR donor to rDNA locus | This study |
| pUC19-rDNA-crtE-crtI-crtB | pUC19-rDNA-hisG-URA3-hisG vector containing *crtE*、*crtB* and *crtI* | This study |
| pMD18T-HisG-NatR-HisG | NHEJ vector with no gene, Nat^R^ | This study |
| pMD18T-HisG-URA3-HisG | NHEJ vector with no gene | This study |
| NHEJ-HMGR-AtoB | NHEJ vector containing *HMGR* and *AtoB* genes | This study |
| NHEJ-AtoB-ERG13 | NHEJ vector containing *ERG13* and *AtoB* genes | This study |
| NHEJ-crtI | NHEJ vector containing *crtI* gene | This study |
| pINA1269 | *Y. lipolytica* integrative plasmid with P_hp4d_ and T_XPR2_，Amp^R^ | ^[3]^ |
| pINA1269-IDI | pINA1269 vector containing *IDI* gene | ^[4]^ |
| pINA1269-IDI-IDI | pINA1269 vector containing two *IDI* genes | ^[4]^ |
| pINA1269-ERG12 | pINA1269 vector containing *ERG12* gene | ^[4]^ |
| pINA1269-HMG1-ERG12 | pINA1269 vector containing *ERG12* and *HMG1*genes | ^[4]^ |
| pINA1269-ERG19 | pINA1269 vector containing *ERG19* gene | ^[4]^ |
| pINA1269-ERG20 | pINA1269 vector containing *ERG20* gene | ^[4]^ |
| pINA1269-ERG13 | pINA1269 vector containing *ERG13* gene | ^[4]^ |
| pINA1269-AMPD | pINA1269 vector containing *AMPD* gene | ^[5]^ |
| pINA1269-ERG20-L-GGS1 | pINA1269 vector containing fusion genes of *ERG20* and *GGS1* | This study |
| pINA1269-ERG20-ERG12 | pINA1269 vector containing *ERG20* and *ERG12* genes | This study |
| pINA1269-ACC1 | pINA1269 vector containing *ACC1* gene | ^[6]^ |
| pINA1269-DGA1 | pINA1269 vector containing *DGA1* gene | ^[6]^ |
| pINA1269-GPD1 | pINA1269 vector containing *GPD1* gene | This study |
| pINA1269-ACC1-DGA1 | pINA1269 vector containing *ACC1* and *DGA1* genes | This study |
| pINA1269-OLE1 | pINA1269 vector containing *OLE1* gene | This study |
| pINA1269-HMG1-ERG12 | pINA1269 vector containing *HMG1* and *ERG12* genes | ^[4]^ |
| pINA1312-DGA1 | pINA1312 vector containing *DGA1* gene | This study |
| pINA1312-DGA1-GGS1 | pINA1312 vector containing *DGA1* and *GGS1* genes | This study |

Table S3 Primers used in this study

| Primers | Sequence |
| --- | --- |
| HMG1-F | AAGAATCATTCAAAGGCGCGCATGCTACAAGCAGCTATTGG |
| HMG1-R | AACTAATTACATGAGGCTAGCCTATGACCGTATGCAAATAT |
| GGS1-F | AAGAATCATTCAAAGGCGCGCATGGATTATAACAGCGCGGA |
| GGS1-R | AACTAATTACATGAGGCTAGCTCACTGCGCATCCTCAAAGT |
| CrtI-F | AAGAATCATTCAAAGGCGCGCATGAAGAAAACCGTGGTGAT |
| CrtI-R | AACTAATTACATGAGGCTAGCTTATTGGAGGTCCTCAATCA |
| CrtE-BDH-F | ATGTCAAAGTGAAGAAATTCAAGCTAGCTTATCGATACGCGT |
| CrtE-BDH-R | TTCATGCACCACTGGAAGATCTGGAATTCCATCTCACTTGCGTATGTAT |
| CrtB-BDH-F | TACGCAAGTGAGATGGAATTCGCTAGCTTATCGATACGCGT |
| CrtB-BDH-R | TTCATGCACCACTGGAAGATCTGCATCTCACTTGCGTATGTAT |
| CrtI-BDH-F | TTTCCATACATACGCAAGTGAGATGGAGTACTAGTGGTACCCGAATTCCTGAGGT |
| CrtI-BDH-R | CACGCGTATCGATAAGCTAGCGCAAATTAAAGCCTTCGAGCGTCCCAAAAC |
| CrtE-YZ-F | ATGGTGTCCGGCTCCAAGGC |
| CrtB-YZ-R | CTAGACAGGTCGCTGCCACA |
| HMGR-BDH-F | TCAACCAAGCGCGGGTAAGCTTGGTACCCGCATTCCTGAG |
| HMGR-BDH-R | GTACTGAGAGTGCACCATATGCAAATTAAAGCCTTCGAGCGT |
| AtoB-BDH-F | GAAGGCTTTAATTTGACTAGTGGTACCCGCATTCCTGAGG |
| AtoB-BDH-R | GTACTGAGAGTGCACCATATGCAAATTAAAGCCTTCGAGCGT |
| NatR-BDH-F | ATTGACTGGAACAGCGGTACCGCTAGCTTATCGATACGCGT |
| NatR-BDH-F | CCACTGGAAGATCTGGGATCCCATCTCACTTGCGTATGTATGG |
| HMGR-YZ-F | GATCCTGGGAGTCTCTACCG |
| AtoB -YZ-R | TGCTGCGAGTCAATCTTGGC |
| CrtI-BDH-F | TCAACCAAGCGCGGGTAAGCTTGGTACCCGCATTCCTG |
| CrtI-BDH-R | GTACTGAGAGTGCACCATATGCAAATTAAAGCCTTCGAGCGT |
| ERG20-F | CAACCACACACATCCACGTGATGTCCAAGGCGAAATTCGA |
| ERG20-R | GCTGCCACCGCCACCGCTGCCACCGCCACCCTACTTCTGTCGCTTGTAAATCTTG |
| GGS1-F | GGTGGCGGTGGCAGCGGTGGCGGTGGCAGCATGGATTATAACAGCGCGGA |
| GGS1-R | CAAGTTCCGTAGTTGGATCCTCACTGCGCATCCTCAAAGT |
| ERG12-F | GCGCGAGGCAGCAGATCCGCACCGCCGCCGCAAGGAAT |
| ERG12-R | CGCGGCCGCATAGGCCACTAGTACACGGGCATCTCACTTGCA |
| GPD-F1 | CAACCACACACATCCACGTGATGAGCGCTCTACTTAGATC |
| GPD-R1 | CTTGGCGACGGTGGTTCCCCAGTTACCAGAACCAACAACG |
| GPD-F2 | CGTTGTTGGTTCTGGTAACTGGGGAACCACCGTCGCCAAG |
| GPD-R2 | CAAGTTCCGTAGTTGGATCCCTAGTTGGCGTGGTAAAGAA |
| DGA-BDH-F | GCGCGAGGCAGCAGATCCGCACCGCCGCCGCAAGGAAT |
| DGA-BDH-R | CGCGGCCGCATAGGCCACTAGTACACGGGCATCTCACTTGCA |
| OLE-F | ACCACACACATCCACGTGATGGTGAAAAACGTGGACCAAG |
| OLE-R | TTCCGTAGTTGGATCCCTAAGCAGCCATGCCAGACA |
| DGA-F | CAACCACACACATCCACGTGATGACTATCGACTCACAATACTACAA |
| DGA-R | AGGCCATGGAGGTACCGGATCCTTACTCAATCATTCGGAACTCTGGG |
| GGS-BDH-F | CAAGTGAGATGCCCGTGTCCGGTACCCGCATTCCTGAGGT |
| GGS-BDH-R | TGTTACACATGGAATTCCAAATTAAAGCCTTCGAGCGTCC |

Figure S1 Lycopene standard curve. Linear range of the standard curve is 25 mg/L, the standard lycopene samples are prepared by running the same extraction process as the samples.


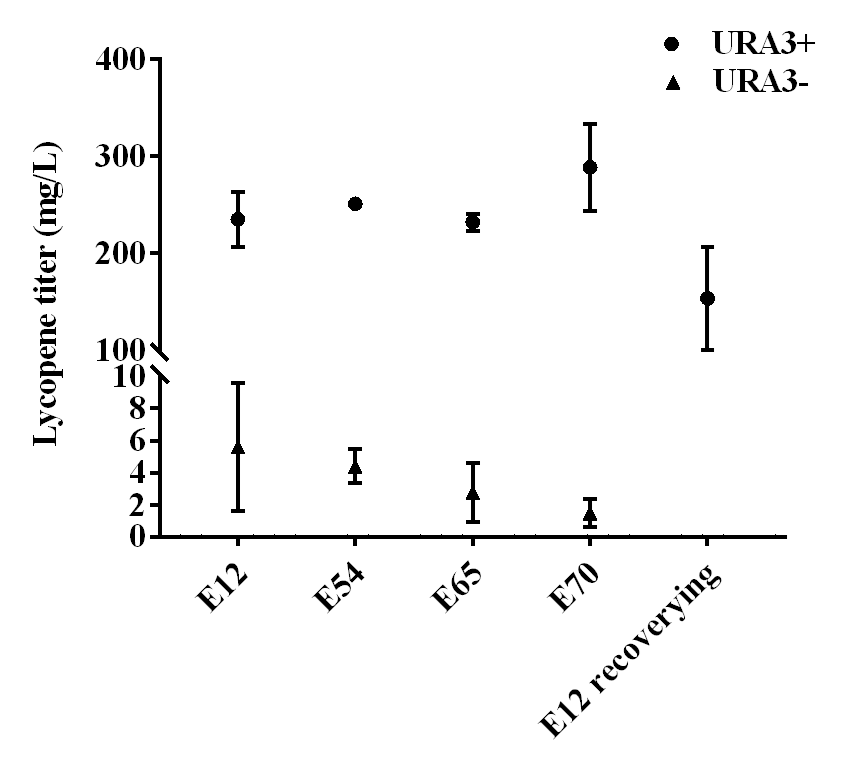


Figure S2 Comparison of lycopene production before and after recovering the selection marker URA3. The uracil auxotrophic marker of four strains with relatively high lycopene production were recycled and the auxotroph was alleviated in the background strain E12 by blank plasmid pINA1312.


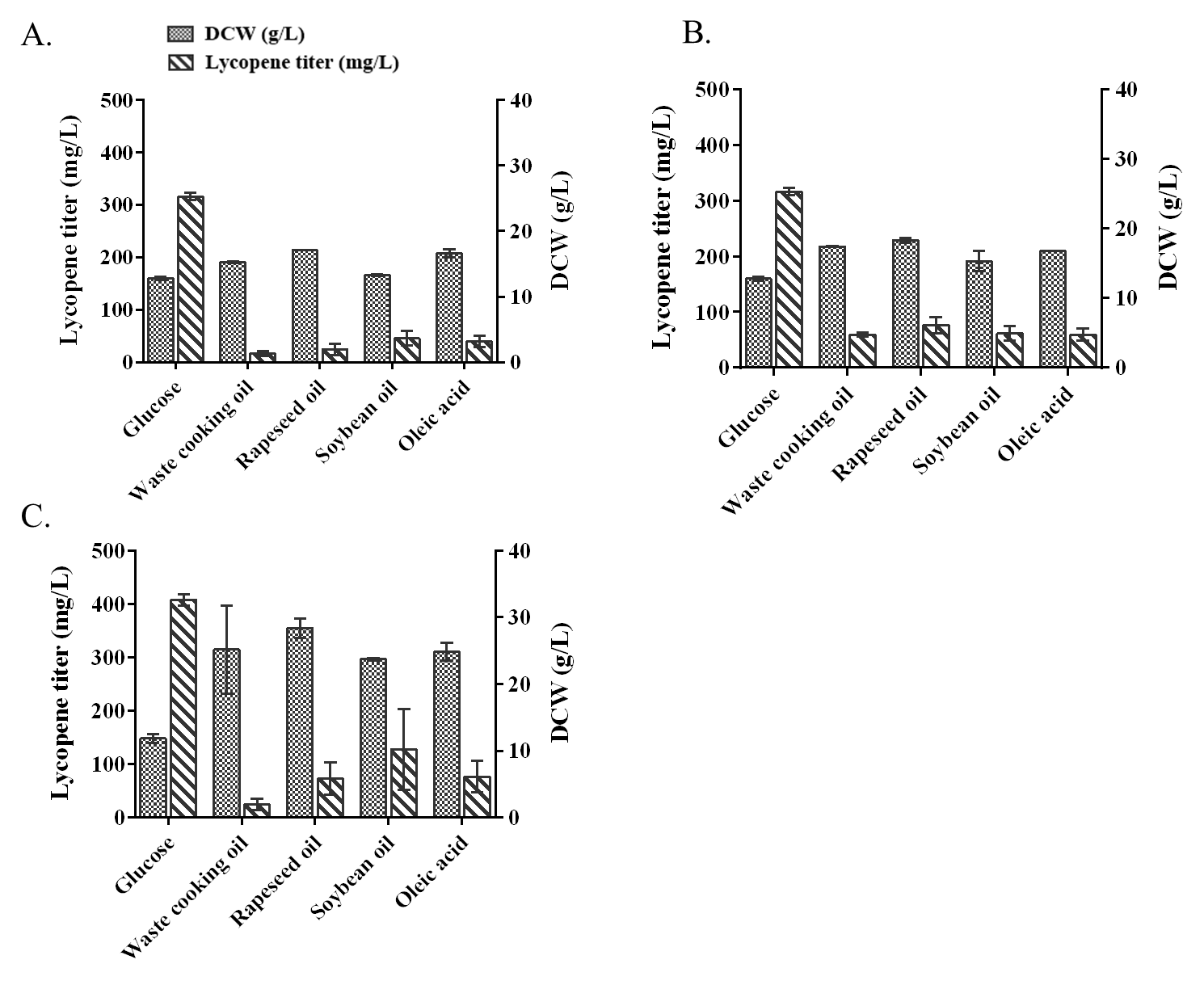


Figure S3 Effects of different oil substances on strain growth and lycopene yield. (A)The performance of strain A7 when different oils were added as the sole carbon source. (B) The performance of strain A7 when different oils were added as the auxiliary carbon source. (C) The performance of strain A7-12 when different oils were added as the auxiliary carbon source.

Figure S4 Effects of organic acids on lycopene yield. Malate, pyruvate, acetate, and citrate (concentration from 0 to 500 mg/L) were chosen as the auxiliary carbon source to boost the lycopene production of in shake flasks.


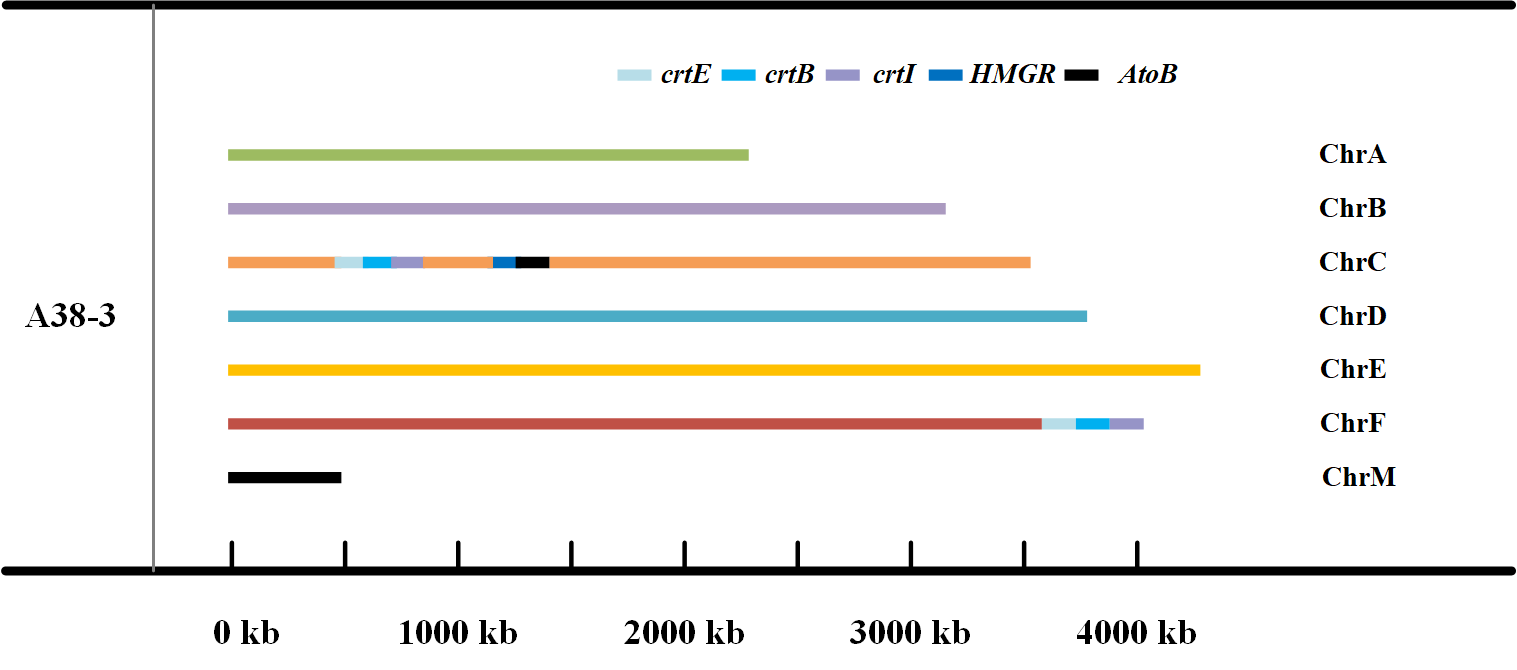


Figure S5 Whole genome sequencing demonstrated random and scattered insertions of *CrtE*/*CrtB*/*CrtI* and *AtoB*/*HMGR* by rDNA and NHEJ-mediated multi-round iterative transformation. Abbreviations: ChrA, chromosome A; ChrB, chromosome B; ChrC, chromosome C; ChrD, chromosome D; ChrE, chromosome E; ChrF, chromosome F; ChrM, chromosome mitochondrion.

**References**

[1] Nicaud JM. Yarrowia lipolytica. Yeast, 2012, 29(10):409-418.

[2] Schwartz C, Shabbir-Hussain M, Frogue K, Blenner M, Wheeldon I. Standardized markerless gene integration for pathway engineering in *Yarrowia lipolytica*. ACS Synth Biol, 2017, 6(3):402-409.

[3] Madzak C, Gaillardin C, Beckerich JM. Heterologous protein expression and secretion in the non-conventional yeast *Yarrowia lipolytica*: a review. J Biotechnol, 2004, 109(1-2):63-81.

[4] Cao X, Lv YB, Chen J, Imanaka T, Wei LJ, Hua Q. Metabolic engineering of oleaginous yeast *Yarrowia lipolytica* for limonene overproduction. Biotechnol Biofuels, 2016, 9:214.

[5] Zhang XK, Nie MY, Chen J, Wei LJ, Hua Q. Multicopy integrants of crt genes and co-expression of AMP deaminase improve lycopene production in *Yarrowia lipolytica*. J Biotechnol, 2019, 289:46-54.

[6] Gao Q, Cao X, Huang YY, Yang JL, Chen J, Wei LJ, Hua Q. Overproduction of fatty acid ethyl esters by the oleaginous yeast *Yarrowia lipolytica* through metabolic engineering and process optimization. ACS Synth Biol, 2018, 7(5):1371-1380.
